# Frequency of *KRAS* and *BRAF* mutations in colorectal carcinoma and their association with clinical-pathological characteristics in a tertiary hospital in Kenya

**DOI:** 10.3389/fmed.2024.1433120

**Published:** 2024-09-19

**Authors:** Samuel Gakinya, Allan Njau, Abdulkarim Abdallah, Ancent Nzioka, James Ogutu

**Affiliations:** ^1^Department of Pathology, Aga Khan University, Nairobi, Kenya; ^2^Department of Surgery, Aga Khan University, Nairobi, Kenya; ^3^Department of Pathology, Kenyatta University Teaching, Referral & Research Hospital, Nairobi, Kenya; ^4^Department of Microbiology, School of Medicine, Kenyatta University, Nairobi, Kenya

**Keywords:** *KRAS*, *BRAF V600E*, colorectal carcinoma, Kenya, Africa

## Abstract

**Introduction:**

Colorectal carcinoma is a leading cause of cancer morbidity and mortality globally. Its management includes the use of targeted therapy which require assessment for biomarkers to choose eligible patients. *KRAS* and *BRAF* mutations are biomarkers predictive of response to anti-EGFR therapy. This study aimed at determining the frequency of *BRAF V600E* and *KRAS* exon 2,3,4 mutations in colorectal carcinoma patients at the Aga Khan University Hospital Nairobi, Kenya.

**Methods:**

Study participants were patients who had colectomy for colorectal carcinoma. They were identified from the laboratory information system. The patients age, gender and tumor location were determined from the medical records. The histological diagnosis, pathological tumor and nodal stage were confirmed by examining hematoxylin and eosin-stained slides prepared from the colectomy specimen. DNA was extracted from the specimens using Qiagen QIAamp DNA FFPE Tissue Kit and PCR performed using EntroGen *KRAS/BRAF* mutation analysis kit following manufacturer’s protocol.

**Results:**

One hundred fourteen patients were enrolled. Colorectal carcinoma was significantly more common in males than females. The mean age at diagnosis was 58 years. Majority of the tumors were in the right colon, were of pathological tumor stage T3 and had nodal involvement. Forty six percent (46%) of the cases had *KRAS* mutations while 5.3% had *BRAF V600E* mutation. *KRAS* mutation was associated with a high pathological tumor stage and nodal involvement.

**Conclusion:**

Colorectal carcinoma in our patients is more common in males and tend to occur at a younger age. The patients tend to have a high tumor pathological stage and nodal involvement at diagnosis. The high frequency of *KRAS* exon 2,3,4 mutation and low frequency *of BRAF V600E* mutations is similar to what has been reported in literature.

## Introduction

Colorectal carcinoma is a leading cause of cancer morbidity and mortality worldwide. It is the third most common cancer diagnosis with an incidence of approximately 11% with the incidence higher in the developed than developing countries (1). In Kenya, the incidence is approximately 7.6% (2). The mainstay of management of colorectal carcinoma is surgical excision with or without chemotherapy, targeted therapy, and radiotherapy (3). The type and outcome of treatment is dependent on several pathological characteristics which include grade of tumor, stage of tumor and biomarker status of the tumor.

Cancer biomarkers are biological characteristics of the tumor which can be objectively assessed and are important in prognostication of the tumor and or predicting response to targeted treatment. According to the guideline by the American Society for Clinical Pathology, College of American Pathologists, Association for Molecular Pathology, and American Society of Clinical Oncology on biomarker evaluation in colorectal carcinoma, evaluation of mutation in *BRAF* and *KRAS* genes is recommended (4). *BRAF* is an oncogene that codes for BRAF protein a serine/threonine kinase which is part of the RAS/MAPK signaling pathway which regulates cellular proliferation, differentiation, migration, and apoptosis (5, 6). A gain of function mutation in this gene results in malignant transformation. The most common *BRAF* mutation in colorectal carcinoma is the *BRAF V600E.* It is associated with an inferior overall survival and disease-free survival (7, 8). In addition, it is predictive of resistance to anti-EGFR targeted therapy in colorectal carcinomas with wild type *RAS* (9, 10).

*RAS* genes are a family of oncogenes which include *KRAS, NRAS* and *HRAS* and encode for RAS proteins all of which are GTPases. Like BRAF protein, they form part of the RAS/MAPK signaling pathway which regulates cellular proliferation, differentiation, migration, and apoptosis (5, 6). A gain of function mutation in these genes results in malignant transformation. In colorectal carcinoma, most of the mutations are in *KRAS* codon 12 or 13 (11). *KRAS* mutations result in aberrant activation of the RAS/MAPK pathway and resistance to anti-EGFR targeted therapy (9). *RAS* mutations have also been associated with a poorer prognosis (11, 12).

While the guidelines on colorectal carcinoma management recommend *KRAS* and *BRAF V600E* mutation analysis, this is not routinely performed in our country. As a result, we do not have much data on the frequency of these mutations in our population. Some studies have suggested that there are racial and geographical differences in the frequency of these mutations (13). The objective of this study was therefore to determine the frequency of *KRAS* and *BRAF* mutations in colorectal carcinomas in our institution. In addition, other clinicopathological characteristics including age, gender tumor location and stage were determined and their association with *KRAS* and *BRAF V600E* mutations described.

The study included tissue from resection specimens of patients who had undergone colorectal resection surgery. The diagnosis was confirmed by reviewing the hematoxylin and eosin-stained slides. *KRAS* and *BRAF* mutation status were determined using RT-PCR.

## Methods

The study participants were colorectal carcinoma patients treated at the Aga Khan University hospital, Nairobi from 2012 to 2022. The participants were identified sequentially from the laboratory information system. The study included patients who had their diagnosis of colorectal carcinoma confirmed on review of the hematoxylin and eosin-stained slides and had sufficient neoplastic tissue for DNA extraction. The review was done by the first author who is an anatomic pathologist. Patients whose tissue blocks were not retrievable or did not have enough neoplastic tissue for DNA extraction were excluded.

### Demographic information (age, gender) and tumor location was extracted from the patient records

Hematoxylin and eosin-stained slides were reviewed for tumor histological classification, tumor staging and grading by the first author. Pathological tumor and nodal staging were done following the American Joint Committee on Cancer (AJCC) 8th edition. The tumors were graded in two categories which were well/moderately differentiated tumors (low-grade) category and poorly/undifferentiated tumors (high grade) category as per previously published recommendations (14). Low grade tumors were defined as having equal to or greater than 50% gland formation while high grade had less than 50% gland formation.

DNA was extracted from 5 to 7 FFPE 5 μm thick sections using the QIAamp DNA FFPE Tissue Kit (Invitrogen) following manufacturer’s instructions. DNA concentration (ng/ul) was determined using Thermo Scientific^™^ NanoDrop Lite Spectrophotometer. 15 ng of DNA in a total volume 30 μL total volume was used.

*KRAS* and *BRAF V600E* mutation were determined by real time PCR on Applied Biosystems quant studio^™^ thermal cycler using EntroGen CE-IVD *KRAS/BRAF* Mutation Analysis Kit for Real-Time PCR (exons 2, 3 and 4 of *KRAS and V600E of BRAF*). The cycling parameters were 95°c for 10 min X1, 95°c for 15 s X40, and 60°c for 60 s X40. The interpretation was as manufacturer’s guidelines.

### Data analysis

The data was analyzed using SPSS software version 20. The demographic information and pathological characteristics were described as means, standard deviations and percentages. Association was assessed using *χ*^2^-test. Tests were statistically significant when *p* < 0.05.

### Ethical consideration

Ethical clearance was given by the Aga Khan University Hospital ERC. NACOSTI research permit was obtained. All the patients were anonymized, and the files were password protected.

## Results

### Clinical and pathological characteristics

A total of 114 colorectal carcinomas were included. Majority 70 (61.4%) were males. Most 81 (71.1%) of the patients were between 41 and 70 years of age with a mean of 58 years. Adenocarcinoma not other specified (NOS) was the predominant histology subtype comprising 108 (94.7%), while mucinous adenocarcinoma and squamous cell carcinoma (SCC) were 5 (4.4%) and 1 (0.9%) of cases, respectively. 105 (92.1%) of the tumors were well/moderately differentiated. The right colon was more commonly involved 61 (53.5%). A substantial proportion of participants were diagnosed with pT3 stage tumors 76 (66.7%), and 63 (55.3%) had nodal involvement (Table 1).

**Table 1:** Clinicopathological characteristics of study participants.

Clinicopathological characteristic of participants		(*N* = 114)
		*n* (%)
Age groups	<40	11 (9.6%)
	41–70	81 (71.1%)
	>70	22 (19.3%)
Age, years (Mean/SD)		57.64 (±14.28)
Gender	Male	70 (61.4%)
	Female	44 (38.6%)
Histology subtype	Adenocarcinoma NOS	108 (94.7%)
	Mucinous adenocarcinoma	5 (4.4%)
	Squamous cell carcinoma	1 (0.9%)
Histology grade	Well/Moderately differentiated (Low grade)	105 (92.1%)
	Poor/Undifferentiated (High grade)	9 (7.9%)
Tumor location	Right Colon	61 (53.5%)
	Left Colon	50 (43.9%)
	Rectum	3 (2.6%)
pT-Stage	pT1	2 (1.8%)
	pT2	9 (7.9%)
	pT3	76 (66.7%)
	pT4	27 (23.7%)
pN-Stage	pN0	51 (44.7%)
	pN1	37 (32.5%)
	pN2	26 (22.8%)

### Colorectal carcinoma and gender

Significantly more males had colorectal carcinoma than females (*p* = 0.004). There was no statistically significant difference between males and females with regards to tumor location, pathological tumor stage and pathological nodal stage (Table 2).

**Table 2:** Colorectal cancer characteristics in our study participants by gender.

Colorectal cancer characteristics by gender			
Age distribution by gender			
	Male	Female	*p* value
<40	2 (2.9%)	9 (20.5%)	*p* = **0.004**
41–70	51 (72.9%)	30 (68.2%)	
>70	17 (24.3%)	5 (11.4%)	
Location distribution by gender			
Right colon	38 (54.3%)	23 (52.3%)	*p* = 0.953
Left colon	30 (42.9%)	20 (45.5%)	
Rectum	2 (2.9%)	1 (2.3%)	
pT stage distribution by gender			
pT1	1 (1.4%)	1 (2.3%)	*p* = 0.752
pT2	7 (10.0%)	2 (4.5%)	
pT3	46 (65.7%)	30 (68.2%)	
pT4	16 (22.9%)	11 (25.0%)	
pN stage distribution by gender			
pN0	30 (42.9%)	21 (47.7%)	*p* = 0.642
pN1	25 (35.7%)	12 (27.3%)	
pN2	15 (21.4%)	11 (25.0%)	

Bold values are *p*<0.05.

### *BRAF V600E* and *KRAS* (exon 2,3,4) mutations

There was a low prevalence of the *BRAF V600E* mutation (5.3%) while *KRAS* mutation was high at 52 (45.6%) (Table 3).

**Table 3:** *BRAF V600E* and *KRAS* mutation frequencies.

Mutation status		*n* (%)
*BRAF V600E*	Positive	6 (5.3%)
	Negative	108 (94.7%)
*KRAS* exon 2,3,4	Positive	52 (45.6%)
	Negative	62 (54.4%)

Among those with *KRAS* gene mutations, G12D and G13D were the most common, each observed in 16 (30.8%) of positive cases, followed by G12C and G12R at 5 (9.6%) and 4 (7.7%) respectively, while other mutations were either absent or rare (Table 4).

**Table 4:** *KRAS* gene mutations frequencies.

Gene mutation	Frequency *n* (%)
*G12R*	4 (7.7%)
*G12C*	5 (9.6%)
*G12S*	0 -
*G12V*	4 (7.7%)
*G12A*	0 –
*G12D*	16 (30.8%)
*G13D*	16 (30.8%)
*Q61R*	3 (5.8%)
*Q61H*	2 (3.8%)
*Q61L*	1 (1.9%)
*K117X*	0 –
*A146X*	1 (1.9%)

### Association between *BRAF V600E* mutation and clinicopathological characteristics

There was no significant association between *BRAF V600E* mutation and age (*p* = 0.596), with similar distribution across age groups. However, there was a significant association with gender (*p* = 0.046), with the mutation being more frequent in males.

No significant associations were found with histology subtype, grade, tumor location, pT-stage, or pN-stage (Table 5).

**Table 5:** Association between *BRAF* mutation and clinicopathological characteristics.

Clinicopathological characteristics	*BRAF V600E* (negative)	BRAF *V600E* (positive)	(*p*-value)
Age			
Mean (±SD)	60.67 (±16.57)	57.47 (±14.22)	
<40	11 (10.2%)	0 (0.0%)	0.529
41–70	77 (71.3%)	4 (66.7%)	
>70	20 (18.5%)	2 (33.3%)	
Gender			
Male	64 (59.3%)	6 (100%)	**0.046**
Female	44 (40.7%)	0 (0.0%)	
Histology subtype			
Adenocarcinoma	103 (95.4%)	5 (83.3%)	
Mucinous adenocarcinoma	4 (0.9%)	1 (16.7%)	0.313
Squamous cell carcinoma	1 (0.9%)	0 (0.0%)	
Histology grade			
Well/moderate (low grade)	100 (92.6%)	5 (83.3%)	0.413
Poor/undifferentiated (high grade)	8 (7.4%)	1 (16.7%)	
Tumor location			
Right colon	56 (51.9%)	5 (83.3%)	
Left colon	49 (45.4%)	1 (16.7%)	0.319
Rectum	3 (2.8%)	0 (0.0%)	
pT-Stage			
pT1	2 (1.9%)	0 (0.0%)	
pT2	8 (7.4%)	1 (16.7%)	0.743
pT3	73 (67.6%)	3 (50.0%)	
pT4	25 (23.1%)	2 (33.3%)	
pN-Stage			
pN0	50 (46.3%)	1 (16.7%)	
pN1	35 (32.4%)	2 (33.2%)	0.205
pN2	23 (21.3%)	3 (50.0%)	

Bold values are *p*<0.05.

### Association between *KRAS* mutations and clinicopathological characteristics

There was no significant association between *KRAS* mutation status and age (*p* = 0.596), gender (*p* = 0.129), histology subtype (*p* = 0.630), histology grade (*p* = 0.941), tumor location (*p* = 0.674), or pN stage (*p* = 0.06). A significant association was observed with pT stage (*p* = 0.01). *KRAS* mutation was associated with higher tumor pathological stage (Table 6).

**Table 6:** Association between *KRAS* mutation and clinicopathological characteristics.

Clinicopathological characteristics	*KRAS* (negative)	*KRAS* (positive)	(*p*-value)
Age			
Mean (±SD)	58.73 (±15.40)	56.73 (±13.33)	
<40	6 (9.7%)	5 (9.6%)	0.639
41–70	46 (74.2%)	35 (67.3%)	
>70	10 (16.1%)	12 (23.1%)	
Gender			
Male	42 (67.7%)	28 (53.8%)	0.129
Female	20 (32.3%)	24 (46.2%)	
Histology subtype			
Adenocarcinoma	58 (93.5%)	50 (96.2%)	
Mucinous adenocarcinoma	3 (4.8%)	2 (40.0%)	0.630
Squamous cell carcinoma	1 (1.6%)	0 (0.0%)	
Histology grade			
Well/moderately differentiated (low grade)	57 (91.9%)	48 (92.3%)	0.941
Poor/undifferentiated (high grade)	5 (8.1%)	4 (7.7%)	
Tumor location			
Right colon	35 (56.5%)	26 (50.0%)	
Left colon	25 (40.3%)	25 (48.1%)	0.674
Rectum	2 (3.2%)	1 (1.9%)	
pT-Stage			
pT1	2 (3.2%)	0 (0.0%)	
pT2	9 (14.5%)	0 (0.0%)	**0.01**
pT3	40 (64.5%)	36 (69.2%)	
pT4	11 (17.7%)	16 (30.8%)	
pN-Stage			
pN0	34 (54.8%)	17 (32.7%)	
pN1	17 (27.4%)	20 (38.5%)	0.06
pN2	11 (17.7%)	15 (28.8%)	

Bold values are *p*<0.05.

## Discussion

*KRAS* and *BRAF* mutations are important biomarkers in the management of colorectal cancer as they are not only predictive of response to anti-EGFR therapy but are also of prognostic value (7, 11, 12). There is a paucity of data on the frequency of these mutations in Africa. This study aimed at determining their frequency in colorectal carcinomas diagnosed at a tertiary hospital in Kenya. The mean age of our patients was 58 years with that of males and females being 54 and 60, respectively. This mean age is slightly lower than that reported in Europe and North America (1, 15) while similar to that reported in studies from Kenya, Tanzania, Uganda and Morocco (16–19). While this difference may be due to differences in life expectancy between the different regions, it may also be due to a racial difference (13). Additional studies are required to explore this finding as it is important in the formulation of screening guidelines. In Kenya, while there is no national screening program for colorectal carcinoma, there are guidelines which currently recommend screening from the age of 45 years for low-risk individuals and 40 years for high-risk individuals (20). The guidelines recommend use of stool occult blood and colonoscopy as the screening modalities. High risk individuals are defined as those with strong family history of colorectal carcinoma or history of colon polyps.

The gender distribution of colorectal carcinoma in our study showed a significant bias toward the male gender (*p* = 0.004). Other studies in different parts of the world show similar findings (1, 15, 21). This may be due to a difference in the risk factor profile in males compared to females. Further epidemiological studies on the risk factors in the Kenyan population may be necessary to identify the cause of this disparity.

As expected, most of the carcinomas were adenocarcinomas and were well/moderately differentiated. The right colon was the most common location of malignancy in both genders. This was however not statistically significant. Right sided cancers have been associated with a worse prognosis (22).

Many of our patients had a pT3 and pT4 tumor stage irrespective of gender. This is significant as high tumor stage is associated with poor disease-free survival, high rates of relapse, high nodal stage, distant metastasis, and overall poor survival (23). Slightly more than 50% of our patients had nodal involvement which is a poor prognostic feature and an indication for adjuvant therapy (3). It is therefore important to encourage screening programs and educate the population on the early symptoms of colorectal carcinoma in a bid to diagnose them at an early tumor and nodal stage to improve outcome as well as avoid use of chemotherapy.

The frequency of *BRAF V600E* mutation in our study was 5.3%. The low frequency is similar to that in other studies in North Africa, Iran, and Saudia showed 0, 3.2 and 2.2%, respectively (19, 24, 25). All the *BRAF V600E* positive patients in our study were male. The significance of this finding is, however, not certain due to the low numbers. Studies with larger populations are advised to explore this finding. There was no statistically significant association of *BRAF V600E* mutation with age, stage, grade, or location. The absence of any association in our study may be due to the relatively low numbers.

The frequency of *KRAS* mutation was 46% with G12D and G13D being the most common mutations. This is similar to what has been reported in other studies. Studies performed in North Africa, Middle East and China showed frequencies of 39.5, 42.2 and 45.2%, respectively (19, 25, 26). *KRAS* mutation was significantly associated with a high pathological tumor stage in this study. Similarly, most of *KRAS* mutation positive patients had nodal involvement though this was not statistically significant. High tumor and nodal stage are associated with a poor survival (23). There was no significant association between KRAS and the other clinical pathological characteristics.

In conclusion, colorectal carcinoma in our study cohort is more common in males and tend to occur at a younger age. Most of the participants have a high tumor stage at diagnosis. The frequency of *KRAS* exon 2,3,4 is 46% while *BRAF V600E* is 5.3%. *KRAS* mutation was associated with a high tumor pathological stage. To the best of our knowledge this is the only study on the frequency of *KRAS* and *BRAF* mutation in Kenya.

## Data Availability

The raw data supporting the conclusions of this article will be made available by the authors, without undue reservation.
